# Novel mutations in *ALDH1A3* associated with autosomal recessive anophthalmia/microphthalmia, and review of the literature

**DOI:** 10.1186/s12881-018-0678-6

**Published:** 2018-09-10

**Authors:** Siying Lin, Gaurav V. Harlalka, Abdul Hameed, Hadia Moattar Reham, Muhammad Yasin, Noor Muhammad, Saadullah Khan, Emma L. Baple, Andrew H. Crosby, Shamim Saleha

**Affiliations:** 1 Medical Research, RILD Wellcome Wolfson Centre (Level 4), Royal Devon and Exeter NHS Foundation Trust, Exeter, Devon EX2 5DW UK; 2 Institute of Biomedical and Genetic Engineering (IBGE), Islamabad, 44000 Pakistan; 3 Department of Biotechnology and Genetic Engineering, Kohat University of Science and Technology (KUST), Kohat, Khyber Pakhtunkhwa 26000 Pakistan

**Keywords:** Autosomal recessive anophthalmia and microphthalmia, *ALDH1A3* gene, Mutations, Variants, Exome sequencing, Consanguineous families

## Abstract

**Background:**

Autosomal recessive anophthalmia and microphthalmia are rare developmental eye defects occurring during early fetal development. Syndromic and non-syndromic forms of anophthalmia and microphthalmia demonstrate extensive genetic and allelic heterogeneity. To date, disease mutations have been identified in 29 causative genes associated with anophthalmia and microphthalmia, with autosomal dominant, autosomal recessive and X-linked inheritance patterns described. Biallelic *ALDH1A3* gene variants are the leading genetic causes of autosomal recessive anophthalmia and microphthalmia in countries with frequent parental consanguinity.

**Methods:**

This study describes genetic investigations in two consanguineous Pakistani families with a total of seven affected individuals with bilateral non-syndromic clinical anophthalmia.

**Results:**

Using whole exome and Sanger sequencing, we identified two novel homozygous *ALDH1A3* sequence variants as likely responsible for the condition in each family; missense mutation [NM_000693.3:c.1240G > C, p.Gly414Arg; Chr15:101447332G > C (GRCh37)] in exon 11 (family 1), and, a frameshift mutation [NM_000693.3:c.172dup, p.Glu58Glyfs*5; Chr15:101425544dup (GRCh37)] in exon 2 predicted to result in protein truncation (family 2).

**Conclusions:**

This study expands the molecular spectrum of pathogenic *ALDH1A3* variants associated with anophthalmia and microphthalmia, and provides further insight of the key role of the *ALDH1A3* in human eye development.

**Electronic supplementary material:**

The online version of this article (10.1186/s12881-018-0678-6) contains supplementary material, which is available to authorized users.

## Background

Anophthalmia and microphthalmia are severe congenital developmental defects of the eye. In the clinical context, anophthalmia refers to complete absence of the globe in the orbit, whilst microphthalmia refers to the presence of a small globe within the orbit. Both anophthalmia and microphthalmia are more commonly bilateral, although they can also present unilaterally. These are relatively rare defects, occurring with an estimated combined incidence of 1 in 10,000 live births [1]. Anophthalmia and microphthalmia can occur as isolated malformations, or as part of a syndrome. Both syndromic and non-syndromic forms of anophthalmia and microphthalmia have been associated with autosomal recessive, autosomal dominant and X-linked patterns of inheritance, and display extensive genetic heterogeneity [2]. Mutations in numerous genes including *RAX*, *PAX6*, *SOX2*, *OTX2*, *VSX2*, *RARB*, *BMP7*, *BCOR*, *BMP4*, *FOXE3*, *STRA6*, *SMOC1*, *SHH*, *SNX3*, *MFRP*, *PRSS56*, *GDF3*, *GDF6*, *TENM3*, *C12orf57*, YAP1, *ABCB6*, *ATOH7*, *VAX1*, *NDP*, *ALDH1A3* and *SMARCA4* have all been described in association with microphthalmia, and some, including *RAX*, *PAX6*, *SOX2*, *OTX2*, *RARB*, *BMP7*, *BCOR*, *BMP4*, *FOXE3*, *STRA6*, *SMOC1*, *GDF6* and *ALDH1A3* have also been described in association with anophthalmia [3–5]. *SOX2* mutations are the major single-gene cause of anophthalmia and microphthalmia, accounting for ~ 10–15% of all cases [6]. Mutations in other genes have been shown to account for another ~ 25% of cases of anophthalmia and microphthalmia [7]. In up to 50–60% of cases however, the underlying genetic cause remains undetermined [2, 8].

Mutations in the aldehyde dehydrogenase 1 family, member A3 (*ALDH1A3*) gene have been found in association with autosomal recessive anophthalmia and microphthalmia in individuals of different ethnicities. Notably, mutations of this gene appear to be the major cause of these conditions in consanguineous families of Pakistani origin [3, 6]. The *ALDH1A3* gene encodes a NAD-dependent aldehyde dehydrogenase, which is among one of three retinaldehyde dehydrogenases (the others being *ALDH1A1* and *ALDH1A2*) that play a key role in the biosynthesis of retinoic acid from retinaldehyde. Retinoic acid functions as a ligand for DNA-binding retinoid receptors that directly regulate transcription of specific target genes in the retinoic-acid signaling pathway in vertebrates [9], and promotes neuronal differentiation in the embryonic nervous system [10]. It also has an important function in the normal early embryonic development of ocular and nasal regions [11].

In this study, we identified homozygous novel missense and frameshift sequence alterations in *ALDH1A3* that segregate with the disease phenotype in consanguineous Pakistani families with isolated anophthalmia, and discuss our findings alongside existing literature in this area.

## Methods

### Ascertainment of family

This study was approved by the ethical committee, Kohat University of Science and Technology (KUST; Pakistan), and families were subsequently recruited. Informed written consent was obtained from parents, and consent was obtained on behalf of their children. A consanguineous family, extending over four generations and comprising of 4 living affected and 12 unaffected members, was recruited from the Khyber Pakhtunkhwa region of Pakistan. All the affected individuals were in the fourth generation. Another consanguineous family, extending over two generations with three affected and five unaffected members, was also recruited from same region of Pakistan. Blood samples were collected from affected and unaffected individuals, and all affected individuals were clinically evaluated by an ophthalmologist for obtaining medical and family histories and clinical assessment.

### Genetic studies

**Fig. 1:**
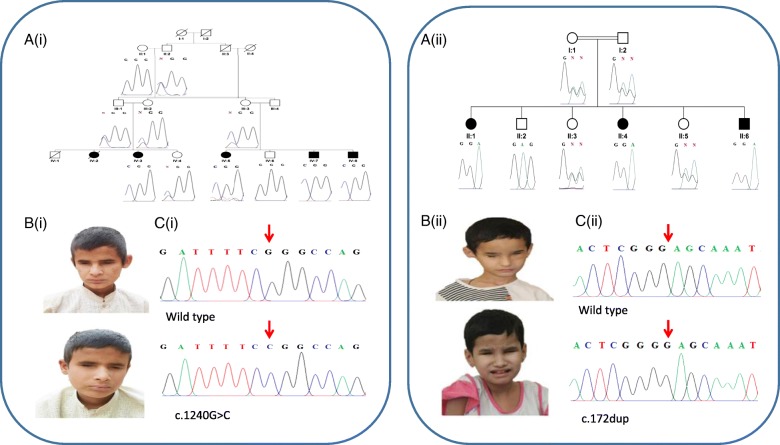
**A** Pedigrees of the Pakistani families investigated, and genetic findings. **A(i)** and **A(ii).** Family 1 (Ai) and family 2 (Aii) pedigrees showing segregation of the variants identified in each case. **B(i)** and **B(ii).** Photographs of two affected individuals in both families with non-syndromic clinical anophthalmia **C(i)** and **C(ii).** Sequence chromatograms showing wild-type alongside *ALDH1A3* [NM_000693.3:c.1240G > C, p.Gly414Arg; Chr15:101447332G > C (GRCh37)] in exon 11 (family 1), and, a frameshift mutation [NM_000693.3:c.172dup, p.Glu58Glyfs*5; Chr15:101425544dup (GRCh37)] variants

Following informed consent, genomic DNA from the blood samples was extracted using the ReliaPrep™ kit (Blood gDNA Miniprep System, Promega) according to the manufacturer’s protocol. To identify the causative gene, whole-exome sequencing was performed on a single affected individual in each family (subject IV: 7 in family 1 and II: 1 in family 2, Fig. 1) to develop a profile of variants not present in publicly available databases and rare sequence variants. Coding regions were captured by HiSeq2000 using paired-end (2 × 100) protocol at a mean coverage depth of 30X at the Otogenetics Corporation (Norcross, GA, USA). The Agilent SureSelect Human All ExonV4 (51 Mb) enrichment kit was used for exome enrichment. The sequence reads were aligned to the human genome reference sequence [hg19] and read alignment, variant calling, and annotation were performed by DNAnexus (DNAnexus Inc., Mountain View, CA; https://dnanexus.com).

Allele-specific primers were designed using Primer3 web software (primer sequences are available upon request) to evaluate segregation of variants via dideoxy sequencing. Polymerase chain reaction (PCR) was undertaken for all recruited family members using allele-specific primers following standard conditions, with products sequenced by Source BioScience LifeSciences (https://www.sourcebioscience.com/). Pathogenicity of the identified missense sequence variation in the *ALDH1A3* gene was also analyzed using PolyPhen-2 (http://genetics.bwh.harvard.edu/pph2/), PROVEAN (http://provean.jcvi.org/index.php) and SIFT (http://provean.jcvi.org/index.php) specialized prediction software. To compare and correlate the *ALDH1A3* gene variants with the phenotype, all reported variants were retrieved from HGMD (http://www.hgmd.cf.ac.uk/ac/search.php), OMIM (https://www.ncbi.nlm.nih.gov/omim/) and PubMed (https://www.ncbi.nlm.nih.gov/pubmed/) databases.

## Results

### Subjects

Pedigree analysis of recruited consanguineous Pakistani families suggested an autosomal recessive inheritance of the disease in these families (Fig. 1). In total, seven living affected individuals with normal intelligence as well as 13 healthy individuals including parents and siblings from both families were investigated. The four affected individuals IV:3, IV:5, IV:7 and IV:8 were 13, 18, 14 and 12 years of age in first family, while three affected individuals II:1, II:4 and II:8 were 19, 10, 4 years of age in second family respectively at the time of examination. On the basis of basic clinical ophthalmic assessment, bilateral isolated anophthalmia was the major finding in all affected members of the investigated families. No other neurological and behavioral features were observed in affected individuals.

### Genetic findings

**Fig. 2:**
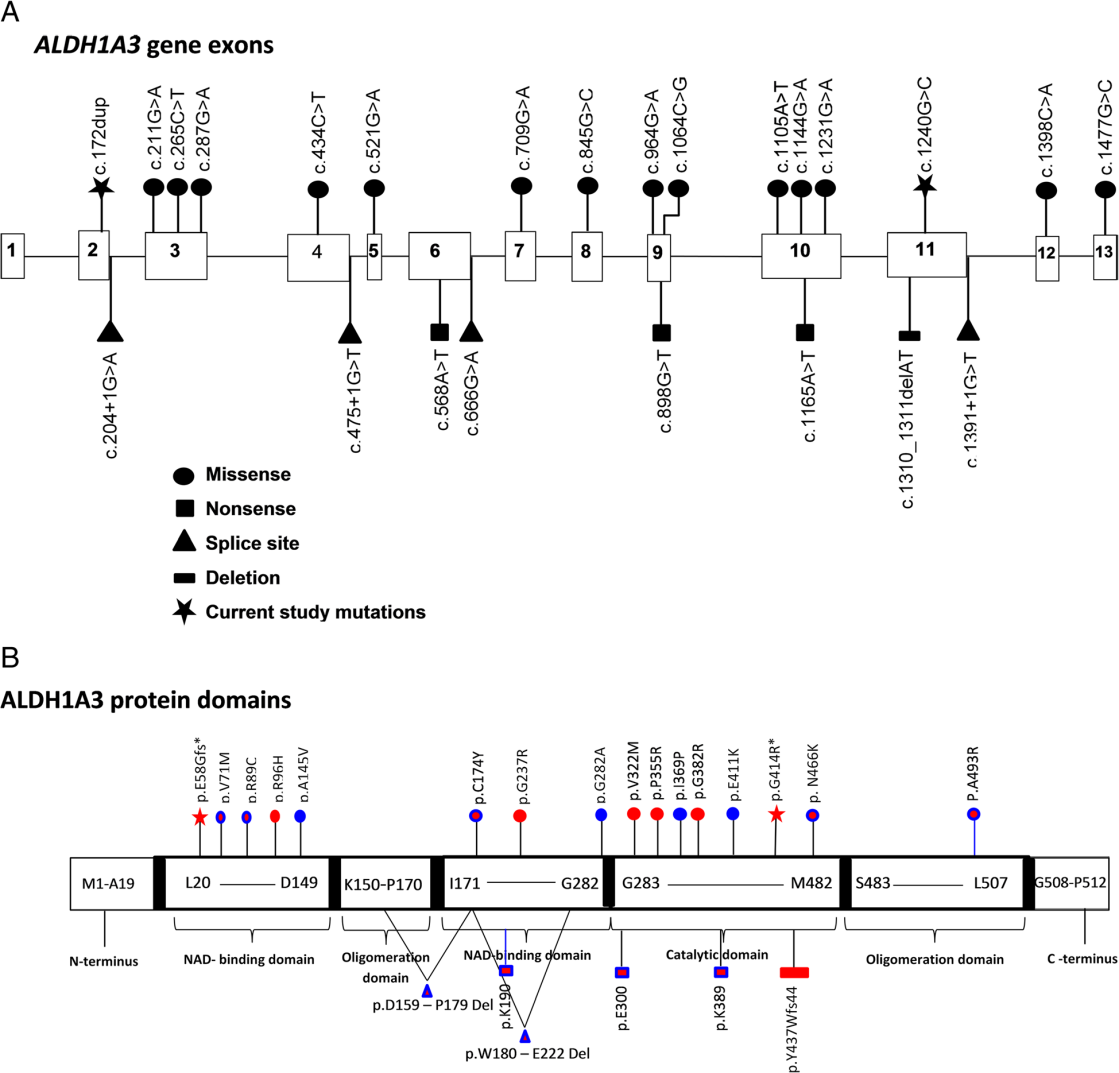
*ALDH1A3* gene mutations associated with anopthalmia and microphthalmia. **a** Schematic representation of exons of the *ALDH1A3* gene highlighting the positions of all disease causing mutations identified to date. **b** Domains of predicted protein product as described by Moretti and colleagues [11], highlighting the positions of all disease associated variants identified to date. Discrete color pattern of variants shows type of phenotype (red: anophthalmia, blue: micophthalmia and a combination of red and blue: both anophthalmia and microphthlamia

The exome data was first inspected to exclude previously described variants in genes known to cause ocular disease. Variants were then assessed and filtered for rare, non-synonymous exonic or splice variants, with a population frequency of < 0.01 in control databases (including the Genome Aggregation Database; gnomAD, the Exome Aggregation Consortium; ExAC, and the 1000 Genomes Project). A single candidate novel homozygous missense variant [NM_000693.3:c.1240G > C; Chr15:101447332G > C (GRCh37)] was identified in exon 11 of *ALDH1A3* (Fig. 1) in first family. This variant leads to a substitution of glycine with arginine and at the evolutionary conserved position 414 (p.Gly414Arg) according to the UCSC Human Genome (GRCh37/ hg19) and Ensemble databases (Additional file 1 A). The p.Gly414Arg variant is not listed in the Genome Aggregation Database (gnomAD). In silico analysis of p.Gly414Arg using PolyPhen-2, PROVEAN and SIFT predicted it to be damaging or deleterious with a score of 1.000, − 7.559 and 3.25 respectively (Additional file 1 B). In family 2, a novel variant [NM_000693.3:c.172dup; Chr15:101425544dup (GRCh37)] was identified in exon 2 of the *ALDH1A3* gene (Fig. 1). This single base pair duplication is predicted to result in a frameshift followed by a premature stop codon (p. Glu58Glyfs*5). The variants in families 1 and 2 segregate as expected for an autosomal recessive condition in each family, and both variants are summarized in Table 1 alongside all other reported disease-associated *ALDH1A3* variants; a schematic representation of each mutation in *ALDH1A3* is also shown in Fig. 2.

**Table 1:** Summary of all reported *ALDH1A3* variants associated with anophthalmia and microphthalmia

Type of mutation	Mutations	Variants	Ethnicity	Patients #	Clinical diagnosis	Literature
Missense	c.211G > A	p. Val71Met	Israeli	9	AM	Mory et al. [15]
	c.265C > T	p. Arg89Cys	Pakistani	2	AM	Fares-Taie et al. [6]
	c.287G > A	p.Arg96His	Chinese	1	A	Liu et al. [21]
	c.434C > T	p. Ala145Val	Saudi Arabian	2	M	Aldahmesh et al. [14]
	c.521G > A	p.Cys174Tyr	Lebanese	3	AM	Roos et al. [16]
	c.709G > A	p.Gly237Arg	Chinese & Iranian	3	A	Liu et al. [21]; Dehghani et al. [19]
	c.845G > C	p.Gly282Ala	Arabic	2	M	Alabdullatif et al. [20]
	c.964G > A	p.Val322Met	Indian	1	A	Ullah et al. [3]
	c.1064C > G	p.Pro355Arg	Egyptian	1	A	Abouzeid et al. [17].
	c.1105A > T	p. Ile369Pro	Saudi Arabian	3	M	Aldahmesh et al. [14]
	c.1144G > A	p.Gly382Arg	Egyptian	4	A	Abouzeid et al. [17]
	c.1231G > A	p.Glu411Lys	Sri Lankan	1	M	Abouzeid et al. [17]
	c.1398C > A	p.Asn466Lys	Turkish	2	AM	Semerci et al. [18]
	c.1477G > C	p. Ala493Pro	Turkish	1	AM	Fares-Taie et al. [6]
	c. 1240G > C	p.Gly414Arg	Pakistani	4	A	Present study
Nonsense	c.568A > T	p.Lys190	Egyptian	2	AM	Yahyavi et al. [5]
	c.898G > T	p.Glu300	Spanish	1	M	Abouzeid et al. [17]
	c.1165A > T	p.Lys389	Hispanic	1	AM	Yahyavi et al. [5]
Splicing	c.204 + 1G > A Alteration of the WT donor site	affecting splicing	Egyptian	2	AM	Abouzeid et al. [17]
	c.475 + 1G > T Skipping of exon 5	p. Asp159-Pro179 del	Moroccan	1	AM	Fares-Taie et al. [6]
	c.666G > A Skipping of exon 6	p.Trp180_Glu222del	Turkish	7	AM	Semerci et al. [18] Plaisancié et al. [13]
	c.1391 + 1G > T Alteration of the WT donor site	affecting splicing	Egyptian	1	A	Abouzeid et al. [17]
Frameshift	c.1310_1311delAT	p.Tyr437Trpfs*44	Pakistani	4	A	Ullah et al. [3]
	c.172dup	p. Glu58Glyfs*5	Pakistani	3	A	Present study

*WT* wild type, *AM* anophthalmia and microphthalmia, *A* anophthalmia, *M* microphthalmia

## Discussion

The *ALDH1A3* (NG_012254.1) gene comprises 13 exons spanning ~ 36 kb on chromosome 15 (15q26.3), encoding a 512-amino acid NAD-dependent aldehyde dehydrogenase localized in the cytoplasm, nucleus, endoplasmic reticulum and mitochondria [12]. Structural analysis reveals that *ALDH1A3* shares high structural homology with other types of aldehyde dehydrogenases. ALDH1A3 assembles as a tetramer, however, each of its monomeric units is independently able to oxidize retinaldehyde into retinoic acid using NAD as a cofactor. Each monomeric unit folds into 13 α-helices, 19 β-sheets and the connecting loops, arranged into three functional domains: the NAD-binding domains (L20-D149 and I171–G282), the catalytic domain (G283–M482), and the C-terminal oligomerization domains (K150–P170 and S483–L507), [11].

The first evidence of involvement of *ALDH1A3* variants in autosomal recessive anophthalmia and microphthalmia in humans was provided by Fares-Taie et al. in 2013 [6]. Since then, mutations of *ALDH1A3* have been identified as a cause of autosomal recessive anophthalmia and microphthalmia in 54 individuals to date. Among these families, 50 individuals are from consanguineous families [3, 5, 6, 13–19], one from a non-consanguineous family, [5] and three are sporadic or individual cases [5, 20]. Recently, Liu and coworkers identified compound heterozygous variants in *ALDH1A3* in a proband from a non-consanguineous family with anophthalmia [21]. Among the reported *ALDH1A3*-associated anophthalmia and microphthalmia cases, 30 have been demonstrated in families of Arab origin including families from Egypt [17], Saudi Arabia [14], Lebanan [16], Morocco [6], Israel [15], and the United Arab Emirates [20], 10 in families of Turkish origin [18], and 12 cases have been found in South and East Asian families including families from Pakistan [3, 6], Iran [19], India [3], Sri Lanka [17] and China [21]. Reported consanguinity rates are high (22–55%) in these populations, which has been associated with an increased risk of autosomal recessive diseases due to homozygosity of regional founder mutations [22]. In European populations, where consanguinity rates are generally less than 0.5% [22], anophthalmia and microphthalmia have been reported much less frequently, with only two cases of Spanish and Hispanic origin reported [5, 17]. Our study, together with previously reported studies, thus provides evidence for the notable occurrence of autosomal recessive anophthalmia and microphthalmia in consanguineous families.

Of the 22 previously reported variants, 14 missense, three nonsense, four splice site variants and one small deletion have been documented. In this study, we report a novel missense mutation (c.1240G > C; p.(Gly414Arg)) in *ALDH1A3* in a consanguineous four generations family of Pakistani origin. This Gly414Arg substitution affects a highly conserved residue across model organisms including humans. This variant, as with the previously documented missense ALDH1A3 variants Val322Met, Ile369Pro, Gly382Arg, Pro355Arg, Glu411Lys and Asn466Lys [3, 14, 17, 18], is presumed to be located in the functionally important catalytic domain that governs substrate specificity. Missense variants in the ALDH1A3 catalytic domain are thought to result in an aberrant tertiary structure with abnormal protein folding, leading to subsequent protein degradation and loss of function, and the novel variant identified in this study is believed to cause disease via a similar mechanism. Two nonsense variants, p.Lys389* and p.Glu300* have also been identified in the catalytic domain of ALDH1A3, resulting in the predicted truncation of the protein product due to mRNA targeted degradation [5, 17]. A single frameshift deletion variant p.Tyr437Trpfs*44 has also been reported in this domain, also predicted to cause loss of function of ALDH1A3 via nonsense-mediated decay [3].

In the oligomerization domain of ALDH1A3, a single missense variant Ala493Pro has been identified, and is expected to hamper the specific activity of the ALDH1A3 tetramer due to the introduction of a helix kink that leads to an incorrect position of the two beta sheets relative to each other within the oligomerization domain at protein level [6]. Fares-Taie et al. [6] found homozygosity for a c.475 + 1G > T splice site mutation in the *ALDH1A3* gene that was predicted to abolish the splice-donor site of intron 4, with an in-frame skipping of exon 5 expected. This would cause a deletion of critical amino acid residues (Asp159-Pro179) in both the oligomerization domain (Asp159-Pro170) and in the NAD-binding domain (Ile171- Pro179) of ALDH1A3 at protein level, presumably affecting both its oligomerization and binding or catalytic abilities. Abouzeid et al. [17] found homozygosity for a c.1391 + 1G > T splice site mutation in the *ALDH1A3* gene that causes alteration of the wild type donor site (http://www.umd.be/HSF3/ or http://krainer01.cshl.edu/cgi-bin/tools/ESE3/esefinder.cgi), (Table 1), and is therefore predicted to affect interaction with core spliceosome proteins resulting in non-functional ALDH1A3 protein production.

Variants in the NAD-binding domain of ALDH1A3 also result in loss of function. The ALDH1A3 variant alleles identified in the NAD-binding domain, important for tetramer stabilization include Val71Met, Arg89Cys, Arg96His, Ala145Val, Cys174Tyr, Gly237Arg and Gly282Ala [6, 14–16, 19–21]. In the present study, a further novel variant (p.Glu58Glyfs*5) was identified in the NAD-binding domain. These variants may impact on tetramer stability, with the newly synthesized unstable proteins predicted to be unstable and therefore subjected to proteasome-dependent degradation in the cells [6, 21]. The Cys174Tyr, Lys190*, Gly237Arg and Gly282Ala variants are located at the foot of the NAD-binding domain (Ile171- Gly282). Variants in this region are important, may directly affect NAD binding by altering the conformation of ALDH1A3 in NAD binding pockets [11], leading to proteasome degradation [5, 21]. A homozygous splice site mutation (c.204 + 1G > A) was found by Abouzeid et al. [17] in the head of NAD binding domain of the ALDH1A3 protein that was predicted to lead to an improperly spliced product by affecting the donor splice site of intron 2. Another homozygous splice site mutation c.666G > A was detected by Semerci et al. [18] in the foot of the NAD-binding domain of the ALDH1A3 protein that was shown to cause an inframe deletion of 43 amino acids (Trp180_Glu222del) at the foot of this domain. These splice site mutations are also likely to affect the tetrameric stability or conformation in NAD binding pockets that are a prerequisite for the normal function of the ALDH1A3 protein.

*ALDH1A3*-associated anophthalmia and microphthalmia, is frequently reported in association with other ocular and extra ocular anomalies, such as the presence of short eyelids, blepharophimosis and reduced palpebral fissures [5, 13, 17, 21], entropion [5], conjunctival symblepharon [17], conjunctival discoloration [17], large eyebrows and synophrys [17, 18], coloboma [5, 14, 16, 17, 20], hypoplasia of the optic tracts and chiasm [5, 6, 15, 17, 18], hypoplastic extra ocular muscles [15, 18], high arched palate [17], refractive errors including both myopia and hyperopia [14, 16], and esotropia [14]. There is a high variability observed in the phenotypic expression of dysmorphic or extra ocular features associated with anophthalmia and microphthalmia, even in individuals with the same *ALDH1A3* genetic variants [13, 18, 19]. Mild hypoplasia of the vermis (variant of Dandy-Walker malformation), as well as pulmonary stenosis and atrial septal defect, have also been reported in association with *ALDH1A3*-associated anophthalmia and microphthalmia [6, 18]. As these extra ocular findings have only been reported in a single individual, it remains unclear if these features are associated with the *ALDH1A3* mutation, or occur due to a separate genetic disorder. Occasionally, patients with *ALDH1A3*-associated anophthalmia and microphthalmia are also reported to have neurocognitive or behavioral features including intellectual disability, developmental delay and autism [6, 14, 16, 18]. However, this association is controversial due to the wide interfamilial variability in the neurocognitive or behavioral outcomes [14, 16, 18], and the important impact of visual impairment during development [23, 24]. In addition, intellectual disabilities due to other genetic disorders may be more common in populations with high consanguinity [25].

It has previously been suggested that the difference in phenotype between microphthalmia and anophthalmia may be the result of residual ALDH1A3 activity [17]. However, a review of all known disease-causing mutations in *ALDH1A3* (Fig. 2 and Table 1) does not seem to support this hypothesis, with no consistent correlation between a particular phenotype (anophthalmia or microphthalmia) and the nature of variation (missense, nonsense, frameshift or splice variants) or the protein domain affected (NAD-binding domain, catalytic domain or oligomerization domain). This may partly be due to difficulty in distinguishing between anophthalmia from severe microphthalmia in routine clinical practice. True congenital anophthalmia can only be diagnosed radiologically or histologically, and most published cases of clinical anophthalmia probably include cases of severe microphthalmia, where residual ocular tissue may have been present in the orbit despite external appearances of an absent globe [1].

There is a wide phenotypic variation in *ALDH1A3*-associated ocular disease. Individuals with the same *ALDH1A3* variant can display both anophthalmia and microphthalmia in different eyes [5, 17, 20], and affected individuals with the same mutation within the same family have been found to have clinical phenotypes of differing severity [5, 13, 16–18]. Epidemiological studies have predicted the contribution of both genetic and environmental factors in the pathogenesis of congenital eye defects including anophthalmia and microphthalmia [26], and the wide phenotypic spectrum seen may result from the impact of other factors such as modifying genes or environmental influences affecting the *ALDH1A3*-associated eye disease phenotype. Further studies would be useful to define this interaction and elucidate underlying pathways.

Determining the underlying diagnosis in patients with anophthalmia and microphthalmia is often challenging due to the genetic heterogeneity of the disorder and the wide variation in phenotypic expression. Taken together, these factors makes it extremely difficult to establish an accurate diagnosis based on clinical presentation alone. This problem is particularly significant in developing countries such as Pakistan, where many families reside in highly remote and rural regions with limited access to healthcare and ophthalmic services. There is also limited availability of specific and expensive radiological investigations such as ocular ultrasound or magnetic resonance imaging which are required in cases of clinical anophthalmia to definitively differentiate between true congenital anophthalmia and severe microphthalmia. This has important prognostic implications, as anophthalmia is more frequently associated with a wide range of systemic anomalies including developmental intracranial and hemifacial anomalies, and as such carries a poorer prognosis than microphthalmia [27]. The application of modern genomic technologies in our families enabled an accurate molecular diagnosis of *ALDH1A3*-associated anophthalmia/ microphthalmia to be established and has facilitated informed genetic counselling. Although extraocular features have been reported in association with *ALDH1A3*-associated ocular disease, these are uncommon, and the associations are controversial, providing a relatively good prognosis for affected families when compared to other known causes of anophthalmia.

## Conclusions

In summary, our results add to the molecular spectrum of autosomal recessive microphthalmia and anophthalmia in general and of Pakistan in particular. The identification of a novel variants in *ALDH1A3* in the present study consolidates the key role of this gene in autosomal recessive anophthalmia and microphthalmia, contributes to the expanding spectrum of disease-causing *ALDH1A3* gene variants, and emphasizes the key function of *ALDH1A3* in human eye development. Given that *ALDH1A3* gene mutations appear to be the most common cause of anophthalmia and microphthalmia in consanguineous families [3, 6, 15–21], screening for variants in this gene before exome analysis in populations with high rates of consanguinity should be considered.

## Additional file

Additional file 1: **A.** Conservation analysis: Multiple alignments of the partial amino acid sequences of ALDH1A3 in a variety of vertebrate and non-vertebrate species, show stringent conservation of Glycine at position 414. **B*.*** In silico analysis of the p.Gly414Arg amino acid substitution identified in exon 11 of the *ALDH1A3* gene. (DOCX 526 kb)
