# Supplementary material for: Novel mutations in ALDH1A3 associated with autosomal recessive anophthalmia/microphthalmia, and review of the literature
Source: BMC Med Genet. 2018 Sep 10;19:160. doi: 10.1186/s12881-018-0678-6 (PMC6131798; doi:10.1186/s12881-018-0678-6)
Supplement: Supplementary file 1 — A. Conservation analysis: Multiple alignments of the partial amino acid sequences of ALDH1A3 in a variety of vertebrate and non-vertebrate species, show stringent conservation of Glycine at position 414. B. In silico analysis of the p.Gly414Arg amino acid substitution identified in exon 11 of the ALDH1A3 gene. (DOCX 526 kb) [file 12881_2018_678_MOESM1_ESM.docx]

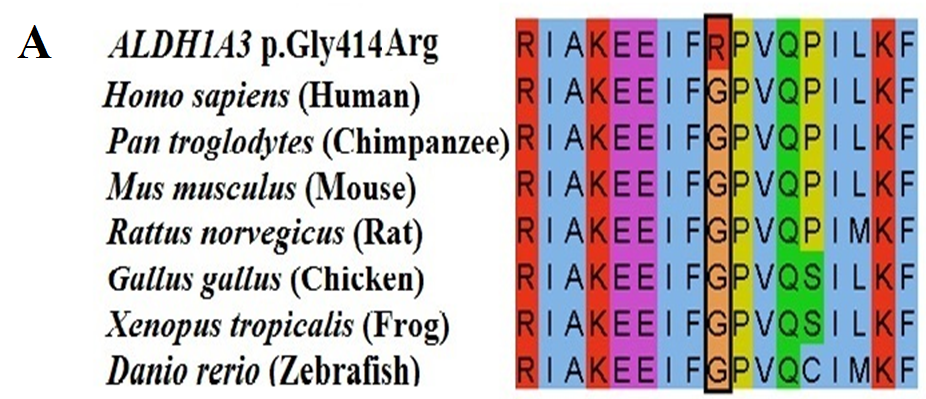


**B**

| **Nucleotide change** | **Residue**  **change** | **Location** | **PolyPhen-2** | | **PROVEAN** | | **SIFT** | |
| --- | --- | --- | --- | --- | --- | --- | --- | --- |
|  |  |  | **Prediction** | **Score** | **Prediction** | **Score** | **Prediction** | **Score** |
| c.1240G>C | p.G414R | Exon 11 | Probably damaging | 1.000 | Deleterious | -7.559 | Damaging | 3.25 |
